# Integration of periodontal pathogens and inflammatory mediators in saliva as biomarkers for periodontitis

**DOI:** 10.3389/fcimb.2026.1846125

**Published:** 2026-07-07

**Authors:** Carl Titusson, Anna Lundmark, Ruben R. G. Soares, Anastasios Damdimopoulos, Gunnar Johannsen, Umear Naseem, Tülay Yucel-Lindberg

**Affiliations:** 1 Department of Dental Medicine, Division of Pediatric Dentistry, Karolinska Institutet, Huddinge, Sweden; 2 Aqua Dental AB, Stockholm, Sweden; 3 Aplex Bio AB, Solna, Sweden; 4 Bioinformatics and Expression Analysis Core Facility, Karolinska Institutet, Stockholm, Sweden; 5 Department of Dental Medicine, Division of Periodontology, Karolinska Institutet, Huddinge, Sweden

**Keywords:** biomarker, host response, Inflammatory mediator, microbiome, periodontitis, saliva

## Abstract

**Background/Objective:**

Periodontitis pathogenesis is driven by oral microbiome dysbiosis and dysregulated host immune responses. This cross-sectional study characterized salivary microbiome and inflammatory mediator profiles to identify candidate biomarkers distinguishing periodontal health from periodontitis (PD) using hyperplex PCR, multiplex assay and exploratory machine learning.

**Materials and methods:**

Stimulated saliva samples from 57 participants (28 periodontally healthy, 29 with PD stage III/IV) were collected and analyzed using Hyperplex PCR (simultaneously amplifying multiple targets in a single assay) for oral bacteria and a multiplex immunoassay including 37 inflammatory mediators. Random forest modeling explored the discriminatory performance of individual and combined biomarkers.

**Results:**

Individuals with PD stage III/IV showed elevated relative abundance of *Filifactor alocis, Fretibacterium* spp.*, Parvimonas micra* (P < 0.05) compared to periodontally healthy individuals. Among determined inflammatory mediators, the levels of Chitinase 3-like 1, sIL-6Rβ, sIL-6Rα, IL-19, pentraxin-3, sTNF-R1 and TWEAK were elevated in PD stage III/IV. Exploratory modeling identified *Fretibacterium* spp. as the strongest individual discriminator (AUC = 0.82), a performance that was not improved by two- or three-marker combinations incorporating additional bacteria or inflammatory mediators.

**Conclusion:**

In exploratory unadjusted analyses, salivary *Fretibacterium* spp. showed the highest discriminatory performance (AUC = 0.82), although this association was not statistically significant after adjustment for age, smoking, and cardiovascular disease. These exploratory findings require further validation in larger, diverse cohorts to assess their potential clinical utility.

## 1 Introduction

In a healthy oral cavity, a delicate equilibrium exists between the host and the commensal microbiota. When this balance is disrupted in a susceptible host, the transition from health to disease can occur. A key driver of this shift is a dysbiotic alteration of the microbial community, triggering a dysregulation in the host’s response and the onset of inflammation (Lasserre et al., 2018). Such microbial imbalance can provoke an exaggerated host response, activating immune cells and the production of inflammatory mediators such as cytokines, chemokines, and protease families like the matrix metalloproteinases (MMPs) (Bascones et al., 2005; Hajishengallis, 2015; Meyle and Chapple, 2015). These processes may collectively lead to progressive loss of connective tissue and alveolar bone ultimately culminating in periodontitis (PD) (Lamont et al., 2018; Curtis et al., 2020). PD affects approximately 31-42% of adults aged ≥ 30 years worldwide (Norderyd et al., 2015; Eke et al., 2020) and is often a silent, progressive condition. Once established, the disease is largely irreversible, underscoring the need for early detection and intervention.

Current diagnostic methods for predicting PD have notable limitations, particularly in sensitivity and specificity (Giannobile, 2012; Slots, 2017). Consequently, research increasingly focuses on identifying potential biomarkers to improve diagnostic accuracy. Saliva, easily collected through non-invasive methods, harbors a wide array of microbial communities as well as inflammatory mediators, cytokines, and proteolytic enzymes. These components may serve as candidate biomarkers for real-time detection of inflammation in oral tissues and ongoing bone degradation (Kinney et al., 2011; Salminen et al., 2014; Lundmark et al., 2017) and represent a potential adjunctive diagnostic method. In this context, numerous publications have explored oral fluids as a promising diagnostic medium (Kinney et al., 2011; Ji and Choi, 2015; Tonetti et al., 2018; Arias-Bujanda et al., 2020). The diagnostic accuracy of salivary single molecular biomarkers for distinguishing between periodontal health and PD in systematically healthy subjects was assessed in a systematic review and meta-analysis published in 2020 (Arias-Bujanda et al., 2020). The salivary biomarkers with good ability to detect PD included MMP-8, MMP-9, Interleukin-1β (IL-1β), IL-6 and haemoglobin (Hb) with reported sensitivity and specificity values: MMP-8 (0.73; 0.71), MMP-9 (0.70; 0.82), IL-1β (0.79; 0.78), IL-6 (0.72; 0.73), Hb (0.72; 0.75) (Arias-Bujanda et al., 2020).

Our research group has previously used 16S rRNA sequencing (Illumina MiSeq) to characterize the salivary microbiome in patients with PD, revealing significantly higher relative abundance of several bacterial taxa compared to periodontally healthy controls (Lundmark et al., 2019). These included *Prevotella* spp., *Phocaeicola* spp., *Fretibacterium* spp., *Treponema socranskii (T.socranskii)*, *Eubacterium saphenum (E.saphenum)*, *Porphyromonas gingivalis (P.gingivalis)*, *Tannerella forsythia (T.forsythia)*, *Filifactor alocis (F.alocis)*, *Fusobacterium nucleatum (F.nucleatum)*, and *Aggregatibacter aphrophilus (A.aphrophilus)*.

Previously we demonstrated that salivary inflammatory mediators including B-cell activating factor (BAFF), soluble IL-6 receptor β (sIL-6Rβ), interferon-β (IFN-β), sIL-6Rα, soluble TNF receptor 1 (sTNFR1), and pentraxin-3 are significantly elevated in patients with PD and/or peri-implantitis compared to periodontally and peri-implant healthy individuals (Titusson et al., 2025). Among these, pentraxin-3 demonstrated the highest diagnostic potential, with an area under the receiver operating curve (AUC) of 0.74 for distinguishing between PD and/or peri-implantitis and healthy controls (Titusson et al., 2025). To date, most studies have evaluated either microbial or host-response markers and only a few have assessed their combined diagnostic value (Salminen et al., 2014; Zhang et al., 2021; Blanco-Pintos et al., 2023; Miller et al., 2025). Moreover, studies simultaneously examining broad microbial and host-response panels remain scarce. Therefore, the aim of this study was to characterize selected salivary periodontitis-associated bacteria and inflammatory mediator profiles in periodontal health and PD stage III/IV, and to explore their potential as candidate diagnostic biomarkers. Using exploratory machine learning approaches (random forest modeling), we evaluated the discriminatory performance of individual bacteria and inflammatory markers, as well as their combinations, to identify non-invasive promising candidates capable of distinguishing PD stage III/IV from periodontal health.

## 2 Materials and methods

### 2.1 Subject sample

A total of 60 patients, recruited from a dental clinic (Stockholm, Sweden) were enrolled in the study. All participants underwent both clinical and radiographic examination. PD was clinically diagnosed based on the following criteria: bleeding on probing >30%, tooth sites with a probing depth ≥6 mm, a clinical attachment level ≥5 mm, and radiographic images (PD stage III/IV, according to 2018 EFP/AAP classification) (Tonetti et al., 2018). Individuals in the periodontally healthy group without periodontal disease exhibited no signs of PD, characterized by the absence of gingival and periodontal inflammation, probing depths ≤3.0mm and no bleeding on probing.

### 2.2 Collection and preparation of saliva samples

Saliva samples were collected and prepared as previously described (Titusson et al., 2025). In summary, participants were asked to refrain from eating or drinking for one hour prior to saliva collection. Saliva samples were collected in the morning, approximately between 9 and 12 during 2018-2019. They proceeded to chew 1.0 g of paraffin wax (Ivoclar Vivadent, Liechtenstein) for 2 minutes, during which the stimulated saliva produced was gathered. Saliva samples were placed into sterile 50mL falcon tubes and immediately frozen at -80 °C. These samples remained frozen until further processing, which involved centrifugation at 500 x g for 10 minutes at 5 °C. The supernatants were then transferred to Eppendorf tubes, stored immediately at -80 °C, and kept for subsequent analyses.

### 2.3 DNA extraction

For DNA isolation, 400 µL of each saliva sample was centrifuged at 10,000 rpm for 15 minutes. The resulting pellets were then resuspended in 200 µL of PBS. Bacterial DNA was extracted from the resuspended pellets using the QIAamp DNA Mini Kit (QIAGEN, Sweden) following the manufacturer’s protocol as previously described (Lundmark et al., 2019). Briefly, the samples 200 µL PBS solution (prepared above) were mixed with tissue lysis buffer and treated with proteinase K (QIAGEN) at 56 °C for 10 minutes. DNA was purified using ethanol-containing buffers and subsequently eluted in 50 µL of nuclease-free water, as per the kit instructions. DNA concentration for each sample was determined using a Qubit™ 2.0 fluorometer (Invitrogen, Life Technologies, USA). DNA could not be extracted from three samples (all from the healthy group), and these were therefore excluded from further analysis, resulting in 57 individuals included in downstream analyses.

### 2.4 Hyperplex PCR panel design for periodontitis-associated bacteria

The Hyperplex PCR (hpPCR) panel targeted 11 periodontal taxa, selected based on established associations with PD and prior sequencing data from our group (Lundmark et al., 2019). The targeted bacteria were *Actinomyces* spp., *A. aphrophilus*, *E. saphenum*, *F. alocis*, *Fretibacterium* spp., *Parvimonas micra (P. micra)*, *P. gingivalis*, *Prevotella denticola (P. denticola)*, *Prevotella intermedia (P. intermedia)*, *T. forsythia*, and *T. socranskii*. All bacterial targets were amplified using a common 16S rRNA amplicon (primers 341F: CCTAHGGGRBGCAGCAG and 518R: TTACCGCGGCTGCTGGC), ensuring standardized detection across taxa. Padlock probe (PLP) design was based on positive and negative selection against target sequences, with any mismatch within the 10−bp region surrounding the ligation site expected to reduce ligation efficiency by ≥100−fold compared with fully complementary sequences. The sequences of the 5′ and 3′ PLP arms for each bacterial target are provided in **Table 1**.Two internal controls were included to create a 13−plex assay. First, an amplicon control PLP targeted a conserved region common to all 16S rRNA sequences within the panel to quantify total 16S rRNA yield; this served as the normalization reference for all bacterial signals. Second, a human RNaseP primer set (RNaseP_F: AGATTTGGACCTGCGAGCG; RNaseP_R: GAGCGGCTGTCTCCACAAGT) and corresponding PLP verified sample integrity. Because padlock−probe chemistry does not accommodate taxon−specific universal controls, normalization to total 16S rRNA provides the most appropriate framework for comparing bacterial relative abundance across samples. Overall, the hpPCR panel represents a focused and biologically relevant subset of the periodontal microbiome suitable for biomarker discovery.

**Table 1:** List of primers and padlock probe (PLP) arms used in the 13-plex hpPCR panel including 11 bacteria targets and 2 controls.

Sequence type	Name	PLP arm	Sequence (5'-3')
Padlock probes	*Actinomyces* spp.	left	pGCTGCATCAGGCTTSCGC
		right	CATCCCTCACGCGRCGTC
	*Aggregatibacter aphrophilus*	left	pGCTATTAACACAACAACCTT
		right	GATTAACGTCAATTTGTTGC
	*Eubacterium saphenum*	left	pTTACAACCCAAAGGCCTTCA
		right	TCTTCCCCTAGGACAGAGGC
	*Filifactor alocis*	left	pACGAATGCCTTCTTCACTCA
		right	CTACTACAGAGTTTTACGAC
	*Fretibacterium* spp.	left	pAAGGCCTTCATCGTTCACGC
		right	TAAAAGAACTTTACAACCCT
	*Parvimonas micra*	left	pCATAGGACAGAGCTTTACGA
		right	TGATACCGTCATTATCTTCT
	*Porphyromonas gingivalis*	left	pTGTCTTCCTTCACGCGACTT
		right	AAGTTTACAATCCTTAGGAC
	*Prevotella denticola*	left	pTCCTGCACGCTACTTGGCTG
		right	TACAACCCRTAGGGCCGTCC
	*Prevotella intermedia*	left	pTCCTGCACGCTACTTGGCTG
		right	ACAACCCATAGGGCCGTCAA
	*Tannerella forsythia*	left	pGTAAAAGAAGTTTACAACCC
		right	GTATCTCATTTTATTCCCCT
	*Treponema socranskii*	left	pCGTTCACGCGGCGTCGCTCC
		right	TACAACCTTCCGGCCTTCTT
	Amplicon control	left	pCGKADTTAGCCGDBVCTT
		right	TTACCGCGGCTGCTGGCA
	RNaseP control	left	pGCCTTCAGGTCAGAACCCGC
		right	TCTCCACAAGTCCGCGCAGA
PCR primers	341F		CCTAHGGGRBGCAGCAG
	518R		TTACCGCGGCTGCTGGC
	RNase P_F		AGATTTGGACCTGCGAGCG
	RNase P_R		GAGCGGCTGTCTCCACAAGT

### 2.5 Analysis of periodontitis-associated bacteria in saliva samples with hyperplex PCR

The relative abundance of selected periodontal bacteria was assessed in saliva samples using custom hpPCR kits (Aplex Bio). PCR amplification was performed by combining 2 µL of extracted nucleic acids with 8 µL of a master mix containing all relevant primers for the targets under analysis. Final primer concentrations were 500 nM for both forward and reverse primers. The mix was then subjected to the following temperatures using a thermal cycler: 25 °C for 2 min, 95 °C for 2 min, followed by 22/25 cycles of [95 °C for 3 sec, 60 °C for 1 min]. After PCR, the amplicons were diluted with 190 µL DNase/RNase-free water and 0.5 µL of the diluted mixture were combined with 19.5 µL of ligation mixture in a separate PCR tube, comprising the mix of PLPs (1 nM each final concentration), ligation buffer and hpPCR Ligase. For PLP ligation, the mixture was placed in the thermocycler and subjected to 95 °C for 20 s and 60 °C for 30 min. 10 µL of rolling circle amplification (RCA) master mix containing hpPCR polymerase were then added to the 20 µL ligation mix and subjected to an additional 2 hours at 37 °C, followed by enzyme inactivation at 60 °C for 20 min. All temperature cycling and incubation steps were performed in a Bio-Rad T100 thermal cycler with default temperature ramp conditions. After RCA, 2.5 µL of the rolling circle products (RCPs) solution was combined with 50 µL of capture buffer containing an RCP reference. The RCP reference serves as an internal control to normalize the capture efficiency of RCPs on the surface. 40 µL of this mix were then transferred into the wells of the slide frame mounted on the capture slide provided in the kit, followed by incubation for 5 min at room temperature (RT). Afterwards, 100 µL of absolute EtOH were added to each well on top of the capture solution and all contents were subsequently discarded. The wells were then washed twice with wash buffer, followed by 50 µL of blocking buffer which and incubated for 5 min at RT, followed by addition of 40 µL of labelling master mix containing the probes. After 60 min incubation at 37 °C, the wells were washed four times with 50 µL wash buffer, the slide frame was disassembled and finally mounted using antifade solution (SlowFade™ Diamond Antifade Mountant, Thermo Fisher Scientific). The images were acquired on a Zeiss Axio Imager 2 with a Hamamatsu Orca Fusion sCMOS camera, Colibri 7 light source, a 20×/0.8 Plan−Apochromat objective, and CHROMA emission filters (525/50, 605/52, 690/50, 785/25). Image acquisition was performed with 9 tiles per sample. Analysis of the raw czi files was performed using a proprietary software tool from APLEX Bio including the following general functions: (1) image cropping and background subtraction, (2) channel alignment, (3) segmentation, (4) spot processing and extraction, (5) Nanopixel decoding algorithm and (6) output of counts per color code normalized to an internal reference. The output data for each sample is the average of two hpPCR technical replicates.

For all target sequences, the signal was corrected (cutoff baseline) using a blank sample (DNase/RNase-free water) measured in quadruplicate. The 341F-518R 16S rRNA amplicon control was used to normalize the output of each bacterial target to the total amount of detectable bacterial DNA in the sample.

### 2.6 Analysis of inflammatory mediators in saliva samples

Stimulated saliva samples (*n* = 57) were assessed for salivary concentrations of inflammatory mediators using a multiplex immunoassay kit panel (Human Inflammation panel, 37-Plex) supplied by Bio-Rad (Bio-Rad Laboratories, Hercules, CA, USA). This pre-mixed wide-ranging multiplex kit consists of 37 inflammation-related biomarkers, including members of the tumor necrosis factor (TNF) superfamily, interferon (IFN), interleukin (IL) superfamily proteins, regulatory T-cell-associated cytokines, matrix metalloproteinases (MMPs) and other immune-modulating proteins. The assay was conducted following the manufacturer’s guidelines and protocols and as described in previous studies (Jansson et al., 2021, 2024). The measured analytes were (limit of detection, LOD, in pg/mL, in square brackets): APRIL (a proliferating ligand, also known as tumor necrosis factor ligand superfamily 13, TNFSF13) [190.0], BAFF [34.7], sCD30 (soluble cluster of differentiation 30 or also known as TNF receptor superfamily 8) [1.0], sCD163 (soluble cluster of differentiation 163) [16.8], chitinase 3-like 1 [10.3], sIL-6Rβ [16.9], IFN-α2 (interferon α2) [0.7], IFN-β [2.0], IFN-γ [0.05], IL-2 [0.1], sIL-6Rα [1.5], IL-8 [2.7], IL-10 [0.6], IL- 11 [0.05], IL-12 (p40) [5.6], IL-12 (p70) [0.1], IL-19 [0.2], IL-20 [3.6], IL-22 [1.1], IL-26 [1.2], IL-27 (p28) [0.1], IL-28A(IFN-λ2) [1.8], IL-29 (IFN-λ1) [1.6], IL-32 [12.3], IL-34 [51.9], IL-35 [3.7], MMP-1 [33.7], MMP-2 [39.7], MMP-3 [28.5], pentraxin-3 [0.8], sTNF-R1 [0.2], sTNF-R2 [3.2], LIGHT (also known as TNFSF14) [10.2], TWEAK (TNF-like weak inducer of apoptosis, also known as TNFSF12) [0.5], Osteocalcin [23.4], Osteopontin [91.3], and TSLP (thymic stromal lymphopoietin) [0.8].

Eleven of the measured analytes, including IL-2, IL-12 (p40), IL-12 (p70), IL-20, IL-26, IL-27, IL-32, IL-34, LIGHT, MMP-2 and MMP-3, had concentrations below the detection limits, and were therefore excluded from further analysis.

### 2.7 Statistical analysis

All statistical analyses were performed in R version 4.5.0 (R Core Team, 2025). Group comparisons based on anamnestic and clinical variables used the Mann-Whitney U test, Fisher’s exact test, or Chi−squared test. Differences in relative abundance of salivary bacteria and levels of salivary inflammatory mediators between PD and periodontally healthy controls were assessed with the Mann–Whitney test. Concentrations of inflammatory mediators were log−transformed to approximate normality, and statistical significance was defined as P < 0.05. To account for multiple testing across univariate biomarker comparisons, P−values were adjusted using the Benjamini–Hochberg false discovery rate (FDR) method with *q* < 0.05 considered significant. Age-, smoking-, and cardiovascular disease (CVD)−adjusted biomarker differences were evaluated using multiple linear regression with periodontal status as the primary predictor. Each confounder was adjusted for individually, following recommended approaches for observational studies investigating multiple risk factors (Gao et al., 2025), and all three were additionally entered in combination to provide a conventionally reported combined estimate. Boxplots were generated in GraphPad Prism 10 (v10.6.0).

Diagnostic performance of single, paired, and triplet biomarker combinations was evaluated using random forest models (*randomForest* v4.7−1.2). Models were trained with ntree = 500, mtry = √p, and nodesize = 1, with trees grown to full depth (i.e., unlimited maximum depth, constrained only by nodesize). Model performance was assessed using manual leave−one−out cross−validation (LOOCV), in which one model was trained per individual, ensuring predictions were generated without data leakage. LOOCV−derived predicted probabilities were used to compute AUCs (*pROC* v1.19.0). Sensitivity and specificity were derived using Youden’s index, and PPV/NPV were calculated using sample prevalence. Circos plots were generated with *circlize* (v0.4.16).

## 3 Results

### 3.1 Characteristics of study cohort

The characteristics of the study cohort, both overall and by periodontal status, are presented in **Table 2**. The participants were distributed between the periodontally healthy group.

**Table 2:** Characteristics of study cohort, in total and divided by periodontal status.

Variable	Total (n=57)	Healthy (n=28)	PD stage III/IV (n=29)	P-value
Age, mean (SD)	51.5 (17.8)	38.5 (11.3)	63.9 (13.5)	**<0.01**
Male / female, *n*	21 / 36	7 / 21	14 /15	0.12
CVD, *n* (%)	7 (12.3)	0 (0)	7 (24.1)	**<0.01**
Diabetes, *n* (%)	1 (1.8)	0 (0)	1 (3.4)	1
MJD, *n* (%)	2 (3.5)	0 (0)	2 (6.9)	0.49
Smokers, *n* (%)	11 (19.3)	1 (3.6)	10 (34.5)	**<0.01**
Bleeding, *n* (%)	16 (28.1)	4 (14.3)	12 (41.4)	**0.038**
Plaque, *n* (%)	11 (19.3)	0 (0)	11 (37.9)	**<0.01**

The Mann Whitney U test, Chi-square test or Fisher’s exact test was used for comparison between groups. P-values in bold indicate statistically significant differences (P <0.05).

PD, periodontitis; SD, standard deviation; CVD, cardiovascular disease; MJD, muscle and joint diseases.

(n=28) and PD stage III/IV group (n=29). The mean age of participants was 51.5 years (± 17.8), and the majority (63.2%) were female. The cohort included individuals with CVD (n=7), diabetes (n=1), and muscle-joint disease (n=2). A minority of participants (19.3%) were smokers. When comparing PD group with periodontally healthy controls, significant differences were observed in age, prevalence of CVD and smoking status (**Table 2**).

### 3.2 Relative abundance of salivary periodontitis-associated bacteria and levels of inflammatory mediators

**Figure 1:**
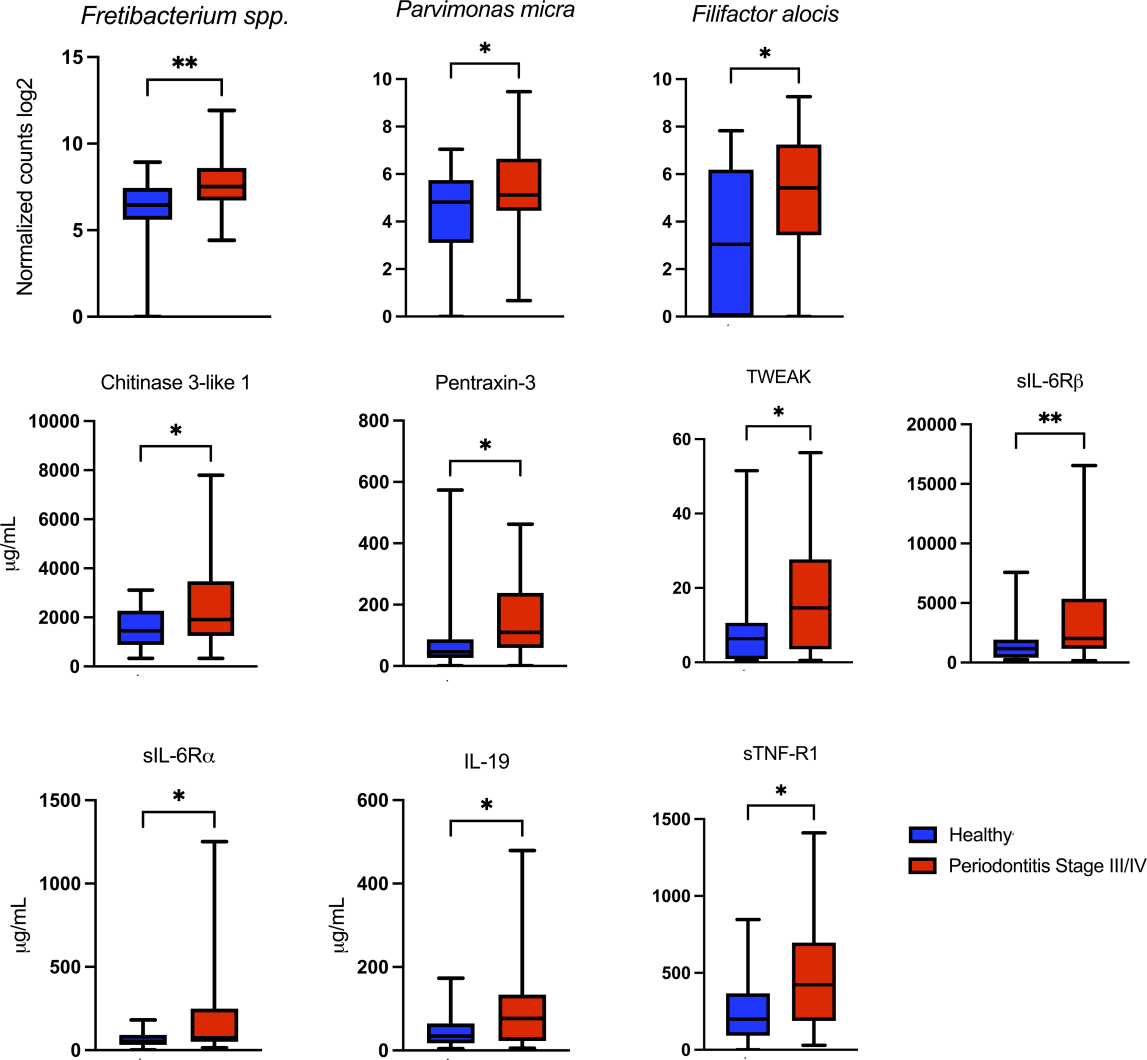
Box plots showing the distribution of statistically significant inflammatory mediator levels and bacterial relative abundance in salivary samples from patients with periodontitis stage III/IV and periodontally healthy controls. Blue boxes representing periodontally healthy controls and red boxes representing patients with periodontitis stage III/IV. *P <0.05; **P <0.01. IL, interleukin; s, soluble; spp., species plural; TNF, tumor necrosis factor; TWEAK, tumor necrosis factor-like weak inducer of apoptosis.

The relative abundance of salivary bacteria and the levels of inflammatory mediators are demonstrated in **Table 3**. Among the investigated bacteria and inflammatory mediators, significant differences between the periodontally healthy and PD stage III/IV groups were observed for three periodontal pathogens and seven inflammatory mediators (**Figure 1**). The relative abundance of *Fretibacterium* spp., *P. micra* and *F. alocis* was higher in the PD stage III/IV group compared to periodontally healthy controls. Furthermore, the levels of inflammatory mediators Chitinase-3-like 1, sIL-6Rβ, sIL-6Rα, IL-19, pentraxin-3, sTNF-R1 and TWEAK were higher in the PD group compared to the periodontally healthy group. To assess biomarker associations with PD while accounting for potential confounding, we performed multiple linear regression analyses with each biomarker as the outcome and periodontal status as the primary predictor, adjusting for age, smoking status, and CVD individually and in combination (**Supplementary Table 1**). Several biomarkers were significantly associated with periodontal status after individual adjustment for age (n=5), smoking status (n=12), and CVD (n=13) (P < 0.05; **Supplementary Table 1**). After adjusting for age, smoking status and CVD in combination, TWEAK levels and the relative abundance of *P. micra* remained significantly elevated in the PD stage III/IV group compared to periodontally healthy controls (P < 0.05; **Supplementary Table 1**). After correction for multiple testing using the Benjamini-Hochberg method, none of the biomarkers reached statistical significance (**Supplementary Table 1**).

**Table 3:** Relative abundance of selected periodontal bacteria and levels of inflammatory mediators in saliva.

Bacteria	Healthy (n=28)	PD stage III/IV (n=29)	P-value
*Actinomyces* spp.	5.86 (2.44)	4.85 (2.42)	0.0847
*Aggregatibacter aphrophilus*	2.11 (1.94)	1.78 (2.14)	0.4544
*Eubacterium saphenum*	0.09 (0.49)	0.54 (1.76)	0.1823
*Filifactor alocis*	3.04 (2.97)	5.03 (2.69)	**0.0116**
*Fretibacterium* spp.	5.92 (2.38)	7.65 (1.54)	**0.0026**
*Parvimonas micra*	4.32 (1.77)	5.37 (1.89)	**0.0487**
*Prevotella denticola*	1.02 (1.28)	0.64 (0.95)	0.2616
*Prevotella intermedia*	2.97 (1.41)	3.45 (1.52)	0.2537
*Porphyromonas gingivalis*	6.01 (2.04)	7.19 (2.17)	0.0778
*Tannerella forsythia*	0.86 (1.45)	2.37 (3.09)	0.1379
*Treponema socranskii*	0.35 (0.92)	0.97 (1.54)	0.1492
Inflammatory mediator			
APRIL (TNFSF13)	69055.51 (57342.89)	100421.57 (133794.54)	0.7016
BAFF (TNFSF13B)	3222.22 (1683.28)	3461.7 (1520.56)	0.4579
sCD30 (TNFRSF8)	10.68 (8.68)	11.8 (8.45)	0.4506
sCD163	1468.37 (1281.66)	2797.16 (3799.47)	0.1460
Chitinase 3-like 1	1601.95 (843.18)	2527.44 (1818.96)	**0.0387**
sIL-6Rβ	1472.29 (1543.72)	4129.06 (4699.25)	**0.0042**
IFN-α2	22.66 (20.83)	23.46 (20.05)	0.4909
IFN-β	39.8 (31.59)	66.53 (58.02)	0.0767
IFN-γ	14.07 (20.71)	13.34 (21.68)	0.7507
sIL-6Rα	67.25 (48.82)	193.37 (283.47)	**0.0293**
IL-8	1004.69 (1116.45)	1525.01 (1566.41)	0.1625
IL-10	3.66 (2.56)	2.81 (1.92)	0.1923
IL-11	0.85 (1.05)	1.18 (2.44)	0.9427
IL-19	47.68 (41.71)	95.54 (98.35)	**0.0403**
IL-22	5.86 (8.46)	6.34 (7.17)	0.3495
IL-28A (IFN-λ2)	9.42 (9.81)	10.03 (10.23)	0.3372
IL-29 (IFN- λ1)	13.89 (24.66)	16.52 (31.55)	0.2401
IL-35	88.97 (99.59)	82.11 (54.95)	0.4018
MMP-1	220.6 (276.71)	265.89 (247.15)	0.1763
Osteocalcin	76.14 (43.16)	86.56 (54.03)	0.2387
Osteopontin	256.44 (205.66)	289.69 (193.96)	0.3833
Pentraxin-3	77.38 (108.34)	157.29 (131.89)	**0.0048**
sTNF-R1	258.52 (214.37)	460.13 (370.04)	**0.0249**
sTNF-R2	47.32 (33.5)	135.28 (192.12)	0.1001
TSLP	9.61 (15.46)	8.26 (12.66)	0.6348
TWEAK (TNFSF12)	9.74 (11.93)	18.26 (17.37)	**0.0384**

The Mann-Whitney U test was used. The levels of inflammatory mediators are presented as mean (SD), in pg/mL. The relative abundance of oral bacteria are presented as relative abundance units. P-values in bold indicate statistically significant differences (P <0.05).

PD, periodontitis; APRIL, a proliferating ligand; BAFF, B-cell activating factor; IFN, interferon; IL, interleukin; MMP, matrix metalloproteinase; TNF, tumor necrosis factor, TSLP, thymic stromal lymphopoietin, TWEAK, TNF-like weak inducer of apoptosis.

### 3.3 Diagnostic performance of salivary biomarkers using random forest modeling

The diagnostic performance of single, pair and combinations of salivary bacteria and inflammatory mediators was evaluated using random forest modeling. Results including AUC, sensitivity, specificity, negative predictive value, and positive predictive value for the highest performing single biomarkers and combinations with the highest AUC are presented in **Table 4**. A single marker, *Fretibacterium* spp. (AUC = 0.82) and *Treponema socranskii* (AUC = 0.78) showed the highest diagnostic accuracy (**Table 4A**). For pairs, the combinations of *Fretibacterium* spp. + *A. aphrophilus* (AUC = 0.80) and Osteopontin + *Fretibacterium* spp. (AUC = 0.79) were top performers (**Table 4B**). When evaluating combinations of three biomarkers, the highest AUC values were observed for sTNF-R2 + *Fretibacterium* spp. + *A. aphrophilus* (AUC = 0.82) and IFN-α2 + *Fretibacterium* spp. + *A. aphrophilus* (AUC = 0.82) (**Table 4C**).

**Table 4A:** Diagnostic performance for the five highest-performing individual salivary biomarkers.

Biomarker	AUC	Sensitivity	Specificity	PPV	NPV
*Fretibacterium* spp.	0.82 (0.70–0.94)	0.83	0.76	0.80	0.79
*Treponema socranskii*	0.78 (0.56–1.00)	0.73	0.57	0.73	0.57
IL-22	0.72 (0.58–0.85)	0.52	0.07	0.37	0.12
sCD30/TNFRSF8	0.67 (0.53–0.82)	0.31	0.25	0.30	0.26
*Porphyromonas gingivalis*	0.65 (0.50–0.80)	0.48	0.15	0.38	0.21

**Table 4B:** Diagnostic performance for the five highest-performing combinations of two salivary biomarkers.

Biomarkers	AUC	Sensitivity	Specificity	PPV	NPV
*Fretibacterium* spp. + *Aggregatibacter aphrophilus*	0.80 (0.68–0.93)	0.79	0.82	0.82	0.79
Osteopontin + *Fretibacterium* spp.	0.79 (0.67–0.92)	0.62	0.93	0.90	0.70
Chitinase 3-like 1 + *Fretibacterium* spp.	0.79 (0.67–0.91)	0.69	0.86	0.83	0.73
sTNF-R2 + *Fretibacterium* spp.	0.79 (0.66–0.91)	0.69	0.82	0.80	0.72
sTNF-R1 + *Fretibacterium* spp.	0.78 (0.66–0.90)	0.62	0.86	0.82	0.69

**Table 4C:** Diagnostic performance for the five highest-performing combinations of three salivary biomarkers.

Biomarkers	AUC	Sensitivity	Specificity	PPV	NPV
sTNF-R2 + *Fretibacterium* spp. + *Aggregatibacter aphrophilus*	0.82 (0.70–0.94)	0.79	0.79	0.79	0.79
IFN-α2 + *Fretibacterium* spp. + *Aggregatibacter aphrophilus*	0.82 (0.70–0.94)	0.76	0.79	0.79	0.76
APRIL (TNFSF13) + IFN-α2 + *Fretibacterium* spp.	0.81 (0.70–0.93)	0.72	0.68	0.70	0.70
IL-11 + *Fretibacterium* spp. + *Aggregatibacter aphrophilus*	0.81 (0.70–0.93)	0.83	0.75	0.77	0.81
sTNF-R1 + sTNF-R2 + *Fretibacterium* spp.	0.81 (0.70–0.93)	0.76	0.79	0.79	0.76

The diagnostic performance estimates of individual salivary biomarkers and combinations was assessed using random forest modeling.

AUC, area under the receiver operating characteristic curve; PPV, positive predictive value; NPV, negative predictive value; IL, interleukin; TNF, tumor necrosis factor; IFN, interferon; APRIL, a proliferating ligand.

### 3.4 Correlations between salivary periodontitis-associated bacteria and inflammatory mediators

**Figure 2:**
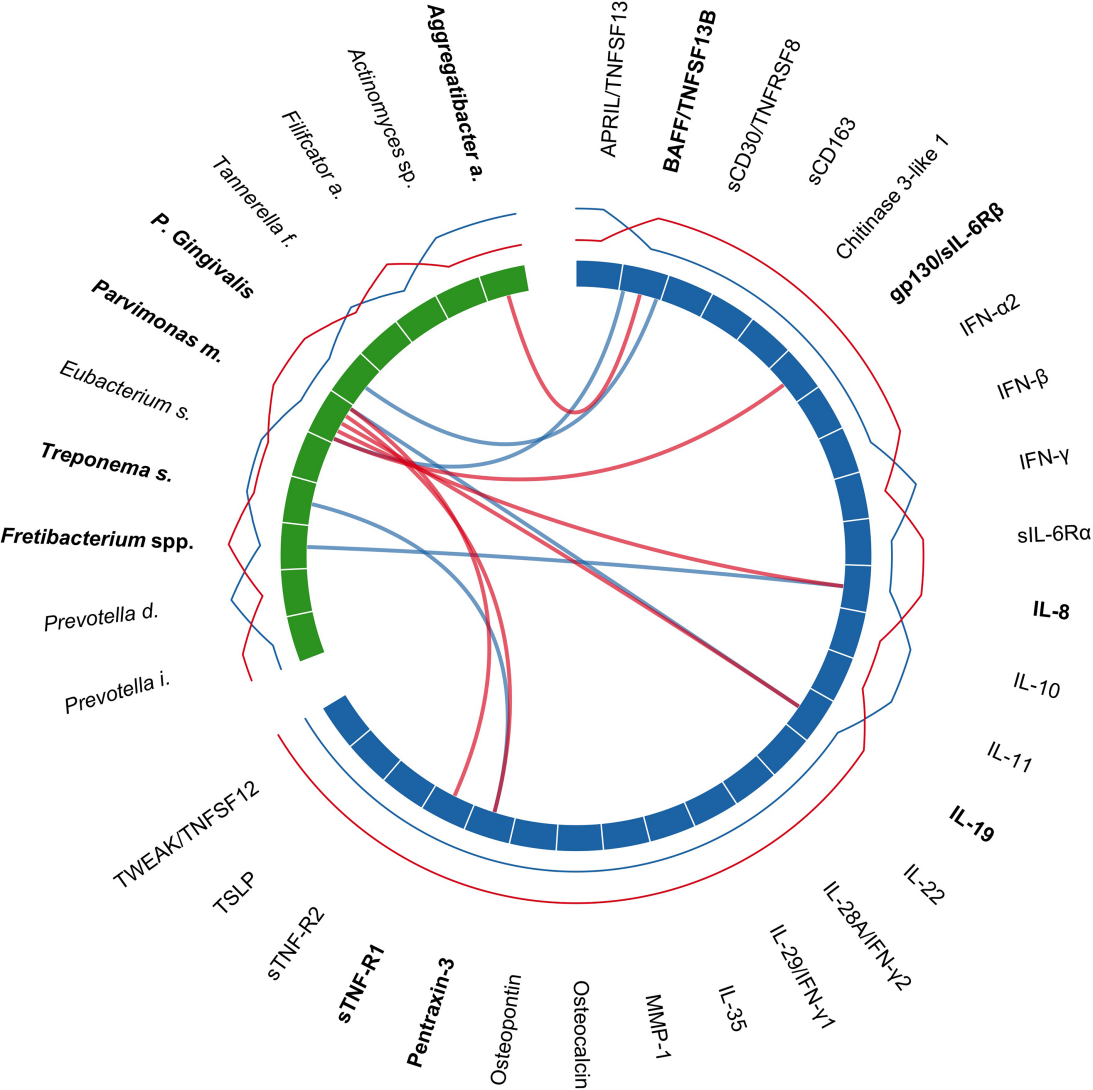
Circos plot showing cross−domain Spearman correlations (|r| ≥ 0.45) between host proteins and bacterial taxa in periodontally health and periodontitis stage III/IV. Blue and red lines represent correlations and median expression patterns in healthy and diseased groups, respectively. Protein and bacterial labels are shown in plain and italic text, with bold labels indicating markers involved in at least one correlation. Outer radial lines depict relative median levels, and inner sector colors distinguish protein (blue) and bacterial (green) domains. APRIL, a proliferating ligand; BAFF, B-cell activating factor; IFN, interferon; IL, interleukin; MMP, matrix metalloproteinase; sCD, soluble cluster of differentiation; sp., species singular; spp., species plural; TNF, tumor necrosis factor; TSLP, thymic stromal lymphopoietin; TWEAK, tumor necrosis factor-like weak inducer of apoptosis.

The correlation between periodontitis-associated bacteria and inflammatory mediators in participants with PD and periodontally healthy controls was analyzed using Spearman correlation and visualized with circos plot (**Figure 2**). Applying a cutoff of |r| ≥ 0.45 identified a number of inflammatory mediator - bacteria correlations in both groups, but the specific associations differed between healthy and diseased status (**Figure 2**). The periodontally healthy controls (blue cords) consisted of several distinct associations spread across multiple taxa, whereas the PD stage III/IV (red cords) involved a different set of inflammatory mediator - bacteria pairs with minimal overlap between conditions. Among the periodontitis-associated bacteria, *P. micra* exhibited the most correlations in the PD group, with associations with five inflammatory mediators: sIL-6Rβ, IL-8, IL-19, pentraxin-3, and sTNF-R1. Notably, the correlations observed for *P. micra* with IL-8 and pentraxin-3 in the PD group differed from those seen in the healthy group, where IL-8 correlated with *Fretibacterium* spp. and pentraxin-3 correlated with *T. socranskii*. Additional differences were observed for BAFF, which correlated with *A. aphrophilus* in PD group, but with *P.gingivalis* and *P. micra* in the periodontally healthy group. Finally, *P. micra* was also correlated with IL-19 in the healthy group.

## 4 Discussion

PD is a chronic inflammatory disease driven by microbial dysbiosis and an exaggerated host immune response. Its asymptomatic progression frequently results in delayed diagnosis until clinically significant periodontal destruction has occurred. Early detection is therefore critical, and identifying reliable, non-invasive salivary biomarkers could significantly improve diagnostic outcomes. Given the complex interplay between bacteria and host immune response, it is important to investigate the presence of both microbial and host-derived proteins as salivary biomarkers. Although previous studies have evaluated combinations of salivary microbial and host-response markers, these investigations have largely been limited to small predefined sets of analytes and have lacked systematic assessment of broader biomarker panels within an integrated modeling framework. To our knowledge, few studies have combined a panel of periodontitis-associated bacteria alongside with a broad range of inflammatory mediators or evaluated their individual and combined diagnostic performance using exploratory machine learning approaches.

In this study, we show that both periodontitis-associated bacteria and inflammatory mediators, when evaluated individually and in combination, exhibit potential for distinguishing periodontal health from PD stage III/IV, with *Fretibacterium* spp. emerging as the strongest discriminatory biomarker, regardless of whether it was evaluated alone or in combination with other markers.

*Fretibacterium* spp., *P. micra*, and *F. alocis* were present at significantly higher abundances in PD (stage III/IV) compared to periodontally healthy controls. Earlier research has shown that *Fretibacterium* spp. are highly discriminative of PD versus health, with their presence being associated with clinical signs of gingival bleeding (Bertelsen et al., 2022; Chen et al., 2022), and several studies have identified this genus as a key taxon enriched in PD (Marchesan et al., 2015; Oliveira et al., 2016; Lundmark et al., 2019; Lafaurie et al., 2022). A higher relative abundance of *P. micra* has likewise been reported in both plaque and saliva samples from individuals with PD compared to healthy controls (Li et al., 2014; Schmidt et al., 2014). Similarly, *F. alocis* has been identified at higher relative abundance in saliva samples from individuals with PD than in healthy controls in multiple studies (Belstrøm et al., 2014; Lafaurie et al., 2022; Lee et al., 2025).

Among the inflammatory mediators, sIL-6Rα, sIL-6Rβ, IL-19, sTNF-R1, TWEAK, Chitinase-3-like 1 and pentraxin-3 were significantly elevated in PD stage III/IV compared to periodontally healthy controls. The soluble IL-6 receptor subunit α and ß have previously been reported by our group to be elevated in saliva samples from patients with PD (Lundmark et al., 2019) as well as in individuals with PD and/or peri-implantitis (Titusson et al., 2025). sTNF-R1 and TWEAK, both members of the TNF superfamily, were also elevated. However, findings on salivary TWEAK in the literature remain inconsistent. For example Yilmaz et al. (2023) reported no significant differences in salivary TWEAK levels between patients with PD and healthy group, either at baseline or follow-up (Yilmaz et al., 2023). Chitinase-3-like 1 and pentraxin-3, both mediators of innate immune responses, have previously been found to be elevated in plasma, serum, and GCF in inflammatory conditions (Pradeep et al., 2011; Ding et al., 2021; Kojima et al., 2022). Our group has reported elevated salivary levels of both markers in patients with PD compared to controls (Lundmark et al., 2019), in individuals with PD and/or peri-implantitis (Titusson et al., 2025), and in individuals with PD and rheumatoid arthritis compared to those with PD alone (Eriksson et al., 2022). Notably, the combination of Chitinase−3−like 1 and *Fretibacterium* spp. yielded an AUC of 0.79, consistent with previous studies reporting positive associations between this glycoprotein and periodontal pathogens, including *Treponema* spp. and *Selenomonas* sp (Lundmark et al., 2019; Eriksson et al., 2022).

Multiple linear regression analyses revealed that the number of significantly associated biomarkers varied considerably depending on the confounder included, with smoking status and CVD yielding more associations than age alone. After combined adjustment, however, only TWEAK and *P. micra* remained significant, and neither retained significance after Benjamini–Hochberg correction. This likely reflects the combined effects of underlying group differences and the limited statistical power inherent to the sample size. Notably, *Fretibacterium* spp. remained significantly associated with periodontal status after individual adjustment for both smoking status and CVD (P-smoking=0.0048, P-CVD=0.0039), and was consistently identified across unadjusted analyses and machine learning models. Together, these findings support *Fretibacterium* spp. as a potential candidate biomarker warranting further investigation in larger, well-powered cohorts.

Exploratory random forest modeling identified that *Fretibacterium* spp. achieved the highest individual discriminatory performance (AUC = 0.82), which was not improved by two- or three-marker combinations, a finding that aligns with Miller et al. (2025), reporting that bacterial markers outperformed individual salivary proteins for distinguishing PD from healthy controls (Miller et al., 2025). *T. socranskii* achieved an AUC of 0.78 in that study (Miller et al., 2025), consistent with our findings. A recent study using machine learning methods identified *Fretibacterium* HMT-362 as a key contributor to model accuracy for distinguishing PD from non-PD (Kageyama et al., 2025). *P.gingivalis* has shown diagnostic potential across multiple studies, with reported AUC values ranging from 0.62 to 0.92, a range that is consistent with our findings (Hyvärinen et al., 2009; Saygun et al., 2011; O'Brien-Simpson et al., 2017). However, some studies report stronger associations when salivary bacteria are combined with inflammatory mediators, compared with either marker type alone (Salminen et al., 2014; Liukkonen et al., 2020).

To explore the host-microbe interactions in relation to periodontal status, we analyzed correlation networks between periodontitis-associated bacteria and inflammatory mediators. The network analyses revealed that the taxa and proteins driving the strongest associations differed between periodontally healthy participants and those with PD stage III/IV, suggesting that the distinct mediator patterns observed in health versus disease underscore the dynamic nature of microbial contributions to periodontal inflammation.

This study has several notable strengths. The simultaneous characterization of both a targeted bacterial panel and a broad profile of inflammatory mediators in the same saliva samples, evaluated within an integrated modeling framework, provides a more comprehensive picture of the host–microbe landscape than studies assessing these components separately. The use of manual LOOCV in the random forest modeling minimized the risk of data leakage, supporting the reliability of the reported AUC values. Additionally, the hpPCR platform enabled sensitive and multiplexed quantification of bacterial relative abundance using existing laboratory infrastructure, representing a practical and clinically translatable approach to targeted microbiome profiling in the context of biomarker discovery. However, several limitations should be acknowledged. The PD and healthy groups differed significantly in age, CVD prevalence, and smoking status, which may have influenced both salivary bacterial and inflammatory mediator profiles. Although multivariable linear regression analyses adjusting for age, smoking status, and CVD were performed, and some biomarkers remained significant after adjustment, none survived correction for multiple testing. Residual confounding cannot be excluded. The relatively small sample size further limits statistical power and contributes to instability in some estimates. Together with the cross-sectional design, which precludes causal inference, these factors limit the generalizability of the findings. An important limitation is that the random forest models, which identified *Fretibacterium* spp. as the top discriminatory marker, were based on unadjusted data and therefore cannot distinguish between the effects of PD and the effects of age, smoking, and CVD which were factors that differed significantly between groups. Accordingly, the results should be considered exploratory and hypothesis-generating rather than confirmatory. Additionally, our findings are limited to the biomarkers examined and should be interpreted cautiously, as inclusion of additional markers not assessed in this study may yield different discriminatory performance. Furthermore, approximately 30% of the measured analytes were excluded due to concentrations below the detection limit, potentially reflecting low endogenous levels and salivary matrix effects, thereby limiting the completeness of the biomarker profile (Chiu et al., 2010; Browne et al., 2013). Consequently, conclusions are based on a subset of detectable biomarkers and should be interpreted accordingly. Furthermore, targeting at the genus level of *Fretibacterium* prevents attribution of diagnostic performance to a specific *Fretibacterium* species. Future studies with larger, diverse cohorts are needed to validate the findings, identify the key *Fretibacterium* species involved, and to evaluate their potential clinical utility.

## 5 Conclusion

Our exploratory study identified salivary *Fretibacterium* spp. as a candidate biomarker in unadjusted analyses and machine learning models, although this association was attenuated after controlling for demographic and clinical confounders. By integrating microbial and host−response markers, this study contributes to a more integrated understanding of the host–microbe landscape underlying periodontal health and disease. These findings highlight the potential of integrated microbiome and host-response profiling in PD, however, validation in larger, demographically balanced cohorts and independent populations, with appropriate adjustment for confounding factors, is required before clinical utility can be established.

## Data Availability

The raw data supporting the conclusions of this article will be made available by the corresponding author upon reasonable request, without undue reservation.
