# Supplementary material for: Integration of periodontal pathogens and inflammatory mediators in saliva as biomarkers for periodontitis
Source: Front Cell Infect Microbiol. 2026 Jul 7;16:1846125. doi: 10.3389/fcimb.2026.1846125 (PMC13384854; doi:10.3389/fcimb.2026.1846125)
Supplement: Supplementary file 1 [file Table1.docx]

**Supplementary material**

| Biomarker | Healthy (N=28) | PD stage III/IV (N=29) | P-age | P-smoking | P-CVD | P-adj | P-FDR |
| --- | --- | --- | --- | --- | --- | --- | --- |
| *Actinomyces* spp*.* | 5.86 (2.44) | 4.85 (2.42) | 0.2728 | 0.1344 | 0.2026 | 0.3327 | 0.2410 |
| *Aggregatibacter aphrophilus* | 2.11 (1.94) | 1.78 (2.14) | 0.1053 | 0.6593 | 0.8434 | 0.2272 | 0.5295 |
| *Eubacterium saphenum* | 0.09 (0.49) | 0.54 (1.76) | 0.4296 | 0.2117 | 0.1498 | 0.3462 | 0.3373 |
| *Filifactor alocis* | 3.04 (2.97) | 5.03 (2.69) | **0.0411** | **0.0249** | 0.1498 | 0.1215 | 0.1071 |
| *Fretibacterium* spp. | 5.92 (2.38) | 7.65 (1.54) | 0.0778 | **0.0048** | **0.0039** | 0.1195 | 0.0597 |
| *Parvimonas micra* | 4.32 (1.77) | 5.37 (1.89) | 0.1447 | **0.0134** | **0.0142** | **0.0394** | 0.1801 |
| *Prevotella denticola* | 1.02 (1.28) | 0.64 (0.95) | 0.7616 | 0.0984 | **0.0458** | 0.2506 | 0.3872 |
| *Prevotella intermedia* | 2.97 (1.41) | 3.45 (1.52) | 0.1619 | 0.2612 | 0.2246 | 0.1825 | 0.3872 |
| *Porphyromonas gingivalis* | 6.01 (2.04) | 7.19 (2.17) | **0.0394** | 0.174 | **0.0283** | 0.0960 | 0.2398 |
| *Tannerella forsythia* | 0.86 (1.45) | 2.37 (3.09) | **0.0221** | 0.2444 | **0.0151** | 0.0957 | 0.3248 |
| *Treponema socranskii* | 0.35 (0.92) | 0.97 (1.54) | 0.9965 | 0.1567 | **0.0316** | 0.9291 | 0.3248 |
| APRIL (TNFSF13) | 69055.51 (57342.89) | 100421.57 (133794.54) | 0.4493 | 0.2315 | 0.1760 | 0.319 | 0.7417 |
| BAFF (TNFSF13B) | 3222.22 (1683.28) | 3461.7 (1520.56) | 0.5499 | 0.2180 | 0.8386 | 0.7919 | 0.5295 |
| sCD30 (TNFRSF8) | 10.68 (8.68) | 11.8 (8.45) | 0.0775 | 0.9320 | 0.9053 | 0.2881 | 0.5295 |
| sCD163 | 1468.37 (1281.66) | 2797.16 (3799.47) | 0.7384 | 0.2237 | 0.1071 | 0.9828 | 0.3248 |
| Chitinase 3-like 1 | 1601.95 (843.18) | 2527.44 (1818.96) | 0.3267 | **0.0041** | **0.0436** | 0.2017 | 0.1655 |
| sIL-6Rβ | 1472.29 (1543.72) | 4129.06 (4699.25) | 0.4503 | **0.0020** | **0.0035** | 0.1718 | 0.0597 |
| IFN-⍺2 | 22.66 (20.83) | 23.46 (20.05) | **0.0402** | 0.7179 | 0.9389 | 0.1521 | 0.5505 |
| IFN-β | 39.8 (31.59) | 66.53 (58.02) | 0.2022 | **0.0166** | 0.1555 | 0.2321 | 0.2398 |
| IFN- γ | 14.07 (20.71) | 13.34 (21.68) | 0.2161 | 0.5392 | 0.9358 | 0.4074 | 0.7716 |
| sIL-6R⍺ | 67.25 (48.82) | 193.37 (283.47) | 0.8288 | **0.0435** | **0.0354** | 0.8866 | 0.1655 |
| IL-8 | 1004.69 (1116.45) | 1525.01 (1566.41) | 0.8736 | **0.0269** | 0.4489 | 0.6729 | 0.334 |
| IL-10 | 3.66 (2.56) | 2.81 (1.92) | 0.4226 | 0.1229 | 0.0812 | 0.7729 | 0.3388 |
| IL-11 | 0.85 (1.05) | 1.18 (2.44) | 0.1166 | 0.5125 | 0.9081 | 0.272 | 0.9427 |
| IL-19 | 47.68 (41.71) | 95.54 (98.35) | 0.2973 | **0.0063** | **0.0299** | 0.1557 | 0.1655 |
| IL-22 | 5.86 (8.46) | 6.34 (7.17) | 0.4747 | 0.7420 | 0.9065 | 0.583 | 0.479 |
| IL-28A/IFN- γ2 | 9.42 (9.81) | 10.03 (10.23) | 0.1134 | 0.7272 | 0.8631 | 0.2873 | 0.479 |
| IL-29/IFN- γ1 | 13.89 (24.66) | 16.52 (31.55) | 0.0734 | 0.8961 | 0.6833 | 0.1598 | 0.3863 |
| IL-35 | 88.97 (99.59) | 82.11 (54.95) | 0.5939 | 0.7054 | 0.5991 | 0.7655 | 0.5126 |
| MMP-1 | 220.6 (276.71) | 265.89 (247.15) | 0.0998 | 0.9505 | 0.6808 | 0.2982 | 0.3373 |
| Osteocalcin | 76.14 (43.16) | 86.56 (54.03) | 0.0500 | 0.9263 | 0.5726 | 0.2451 | 0.3863 |
| Osteopontin | 256.44 (205.66) | 289.69 (193.96) | 0.1286 | 0.8457 | 0.8133 | 0.3495 | 0.5064 |
| Pentraxin-3 | 77.38 (108.34) | 157.29 (131.89) | 0.3436 | **0.0032** | **0.0154** | 0.1299 | 0.0597 |
| sTNF-R1 | 258.52 (214.37) | 460.13 (370.04) | 0.6942 | **0.0041** | **0.0199** | 0.3822 | 0.1655 |
| sTNF-R2 | 47.32 (33.5) | 135.28 (192.12) | 0.9750 | **0.0325** | **0.0195** | 0.8917 | 0.2645 |
| TSLP | 9.61 (15.46) | 8.26 (12.66) | 0.2863 | 0.5732 | 0.7802 | 0.3912 | 0.6908 |
| TWEAK (TNFSF12) | 9.74 (11.93) | 18.26 (17.37) | **0.0095** | 0.1152 | 0.0759 | **0.0412** | 0.1655 |

**Table S1.** Relative abundance of salivary bacteria and levels of inflammatory mediators.

Note: The Mann-Whitney U test was used. The relative abundance of oral bacteria are presented as relative abundance units. The levels of inflammatory mediators are presented as mean (SD) in pg/mL. *p*-values in bold indicate statistically significant differences (P <0.05). The FDR method used was Benjamini-Hochberg. Adjusted p-values were estimated using multiple linear regression, with periodontal status as the primary predictor. Models accounted for age, smoking status and cardiovascular disease (CVD) individually (P-age, P-smoking, P-CVD respectively) and in combination (P-adj).

Abbreviations: PD, periodontitis; APRIL, a proliferating ligand; BAFF, B-cell activating factor; IFN, interferon; IL, interleukin; MMP, matrix metalloproteinase; TNF, tumor necrosis factor, TSLP, thymic stromal lymphopoietin, TWEAK, TNF-like weak inducer of apoptosis.
